# Herpes Simplex Virus Latency: The DNA Repair-Centered Pathway

**DOI:** 10.1155/2017/7028194

**Published:** 2017-02-01

**Authors:** Jay C. Brown

**Affiliations:** Department of Microbiology, Immunology, and Cancer Biology, University of Virginia Health System, Charlottesville, VA 22908, USA

## Abstract

Like all herpesviruses, herpes simplex virus 1 (HSV1) is able to produce lytic or latent infections depending on the host cell type. Lytic infections occur in a broad range of cells while latency is highly specific for neurons. Although latency suggests itself as an attractive target for novel anti-HSV1 therapies, progress in their development has been slowed due in part to a lack of agreement about the basic biochemical mechanisms involved. Among the possibilities being considered is a pathway in which DNA repair mechanisms play a central role. Repair is suggested to be involved in both HSV1 entry into latency and reactivation from it. Here I describe the basic features of the DNA repair-centered pathway and discuss some of the experimental evidence supporting it. The pathway is particularly attractive because it is able to account for important features of the latent response, including the specificity for neurons, the specificity for neurons of the peripheral compared to the central nervous system, the high rate of genetic recombination in HSV1-infected cells, and the genetic identity of infecting and reactivated virus.

## 1. Introduction

All herpesviruses are able to cause both lytic and latent infections. Lytic infection refers to the situation in which the virus replicates in a host cell and causes its lysis, releasing hundreds to thousands of progeny virions. A latent infection is quite different. Here the virus enters into a refractory state in which little or no progeny virus is produced and the cell is not immediately damaged. The virus DNA is present in the latently infected cell nucleus, but there is little DNA replication and only minimal expression of virus-encoded genes. The virus can be reactivated from latency following an appropriate stimulus, however, and reactivation causes lytic virus replication [1–3].

The ability to enter into latency provides herpesviruses with an important survival advantage. In a lytic infection the virus is exposed to components of the immune response that have the potential to clear the virus from the host. In latency, however, infected cells are less readily recognized by the immune system because of the low level of virus gene expression. As a result, the virus can survive an otherwise effective immune response and be reactivated later to spread its infection in a less hostile immunological environment [4].

Herpes simplex virus (HSV1) resembles other herpesviruses in its ability to cause both lytic and latent infections [1, 5]. Lytic infections are produced in epithelial cells of the oral mucosa causing cold sores and other lesions. Progeny virus from this initial infection is able to traffic to sensory neurons in the trigeminal ganglion where a latent infection is produced. Virus reactivated from latently infected neurons migrates back to the site of the initial infection in the oral epithelium producing a second lytic infection. It is usual for a patient to experience many cycles of HSV1 entry into latency and reactivation from it.

The important role of latency in HSV1 pathogenesis has suggested that novel inhibitors targeting latency may be effective as an adjunct to acyclovir for HSV1 therapy. Both entry into latency and reactivation suggest themselves as attractive targets. The pathway to identification of the desired inhibitors would be easier, however, if investigators had a clear understanding of the molecular mechanisms involved in latency. So far, however, despite a large amount of experimental effort and intense interest in the topic, there remains an abundance of viable models for the basic biochemical events involved [1, 5].

Here I describe one of the possibilities, the DNA repair-centered pathway [6]. I begin with a brief summary of HSV1 lytic replication and the basic features of latency. There follows a description of the proposed repair-centered pathway, a summary of the experimental evidence that supports it and an account of how the proposed pathway is compatible with the main features of HSV1 latency and reactivation as they are currently understood.

## 2. Lytic HSV1 Infection

**Figure 1:**
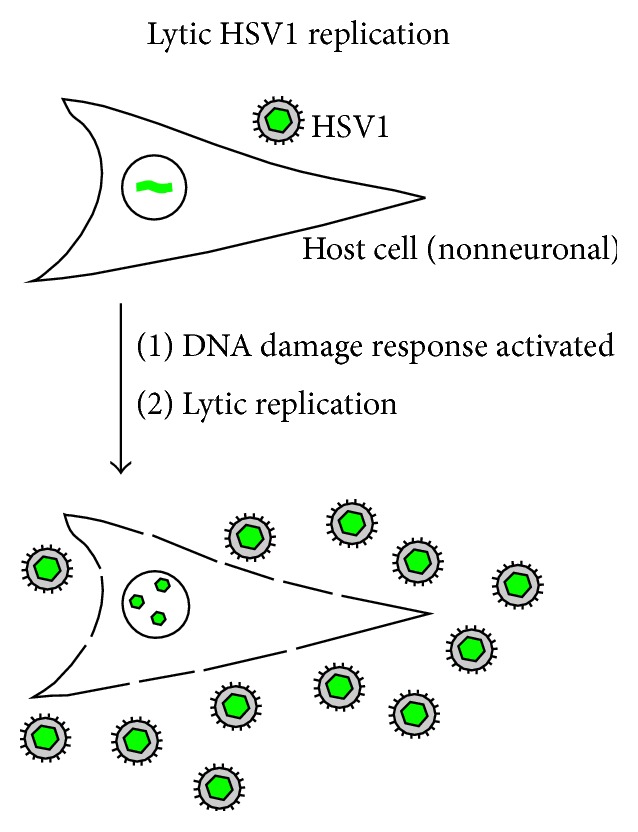
Illustration of lytic HSV1 replication as it is observed in nonneuronal cells. Note that host-encoded DNA damage response proteins are activated following DNA entry into nonneuronal cells and DDR proteins actively potentiate lytic virus growth.

The most common HSV1 infections begin when extracellular virus binds to epithelial cells surrounding the mouth and present in the oral mucosa [4, 5]. Virus binds to receptors on the host cell surface and there follows a fusion event involving host and virus membranes. Fusion results in deposition of the virus nucleocapsid into the peripheral cytoplasm of the host cell. From there it migrates to the cell nucleus, docks at a nuclear pore, and injects the virus DNA into the nucleoplasm. Only the virus DNA enters the nucleus; the parental capsid remains outside. Once inside the nucleus the virus DNA is replicated primarily by the virus-encoded DNA-dependent DNA polymerase. Virus DNA synthesis is also thought to involve activation of cellular components of the DNA damage response (DDR) as described below (see Figure 1) [7, 8]. At the same time, virus genes are expressed beginning with the synthesis of virus-specific messenger RNAs by the host cell-encoded DNA-dependent RNA polymerase.

Assembly of progeny virus begins after sufficient amounts of virus DNA and proteins have been made. Assembly starts in the nucleus with capsid proteins that have been synthesized in the cytoplasm and imported into the nucleus. Capsids are assembled in the nucleus and filled there with virus DNA using a mechanism in which DNA is injected into a preformed capsid [9, 10].

Further assembly steps take place in the host cell cytoplasm [11]. DNA-filled capsids exit the nucleus and acquire tegument and membrane layers in an engulfment event involving vesicles containing the components of both layers. Mature progeny virions then exit the host cell by direct spreading to adjacent cells or as the host cell is lysed. A single cycle of HSV1 replication can take up to 24 hours.

Cold sore lesions require a few days to develop and they can last for a week or more. Spreading of lesions is eventually controlled by an effective immune response involving components of both the innate and acquired responses. Lesions recede without scarring at the site of infection and cold sores do not ordinarily require medical intervention [12].

## 3. Latency and Reactivation from Latency

Latency can be thought of as an extension of a lytic infection or perhaps as a diversion from it. Progeny virus produced at the initial site of infection in the oral epithelium spreads in three different ways: (a) it traffics laterally into adjacent epithelial cells to create a cold sore; (b) it is shed from the skin surface to reach contacts of the infected patient; and (c) it spreads internally to infect adjacent sensory neurons. Initiation of latency involves the third of the above pathways, infection of adjacent neurons [13].

Infection of neurons begins in the same manner as infection of other cell types. HSV1 binds to receptors on the cell surface; a membrane fusion event ensues depositing the nucleocapsid into the peripheral cytoplasm; the nucleocapsid traffics to the nucleus and injects the virus DNA. It is at this point that the infection stalls. Virus DNA synthesis and production of progeny virus are blocked completely creating the latent state. Neurons containing latent HSV1 genomes are concentrated in the trigeminal ganglion as these are prominent among the neurons that innervate the oral epithelium. Latency can persist in the trigeminal ganglion for the lifetime of the patient with the possibility for reactivation at any time. Apart from episodes of reactivation, latent infections do not produce symptoms for the patient [5].

For an infecting HSV1 virion, latency would be a terminal event if it were not for the possibility of reactivation. During latency the virus DNA is present in the neuron cell nucleus, but it has no way to be replicated or to create a lytic infection. Reactivation follows after a stimulus that is not well characterized [14, 15]. Most effective stimuli involve stress to the patient. This can be genotoxic stress such as exposure to sunlight; emotional and physical stress can also initiate reactivation. Once reactivation has been triggered, HSV1 replication follows the same pathway found in lytic infections. Replication occurs first in the neuron; it then spreads by way of the neuron to the original site of infection in the oral epithelium. As in the case of a primary lytic infection, progeny virus arising from a reactivated infection can be spread to the patient's contacts. Also, as in primary infections, symptoms arising from reactivated infections are effectively controlled by the immune response. Most affected patients suffer multiple reactivated infections at intervals of weeks to months.

## 4. The DNA Repair-Centered Pathway for HSV1 Latency and Reactivation: Basic Features of the Pathway

**Figure 2:**
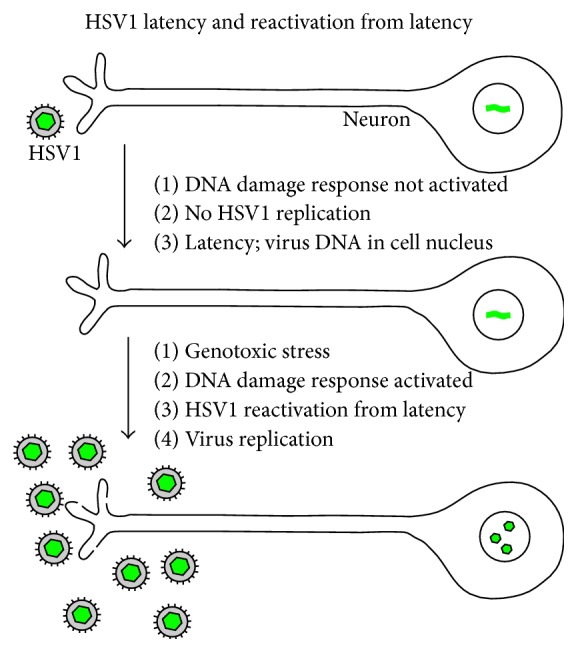
Illustration of HSV1 entry into latency and reactivation as proposed in the DNA repair-centered pathway. Note that DNA damage response proteins are not activated following entry of HSV1 into neurons, a condition that permits virus entry into latency. Note also that reactivation occurs following an accumulation of DNA damage in the latently infected cell. Reactivation results in HSV1 replication in the neuron as illustrated here.

The proposed pathway focusses on the observation that lytic HSV1 replication depends on the activity of cell-encoded DNA repair proteins [16–20]. Components of the DNA damage response, for instance, are required and are available because most cell types respond to HSV1 infection by upregulating and activating the DDR. Required repair activities are found among those involved in mismatch repair and homologous recombination-dependent repair. All are readily available in most epithelial cells due to the cell's continuing need for DNA repair capacity. The same is not true of neurons. HSV1 infection of these cells does not activate the DDR to the same extent found in nonneuronal cells [6, 21, 22]. It is proposed therefore that HSV1 is unable to replicate in mature neurons and enters into latency because DNA repair proteins are not activated in response to infection (see Figure 2).

Reactivation is proposed to be the reverse of entry into latency. It is suggested that an accumulation of damage to both the neuronal and virus DNA eventually reaches a level at which DNA repair pathways are activated. Overall activation includes activation of repair functions required for HSV1 lytic growth, and the neuron becomes permissive for replication. HSV1 replication follows producing progeny virus that is transmitted by way of neurons to the site of the original infection in the oral epithelium (Figure 2). There lesions are produced that follow the same pathway of growth and control by the immune system found in the primary infection.

## 5. Experimental Observations That Support the Repair-Centered Pathway

The DNA repair-centered pathway is supported by experimental studies documenting the following: (1) activation of DNA repair functions following HSV1 infection of nonneuronal cells; (2) the requirement for activation of DNA repair proteins in lytic HSV1 replication; (3) failure of DNA repair protein activation following HSV1 infection of neurons; and (4) reactivation of latent HSV1 from neurons following excess of DNA damage. Studies supporting each of the four conclusions are described briefly below.

### 5.1. DNA Repair Proteins Are Activated following HSV1 Infection of Nonneuronal Cells

Most studies of repair protein activation have been performed with cells in culture. Cells are infected with HSV1 and assayed thereafter by western blot using antibodies specific for the activated form of repair proteins. For instance, in a representative study, infected HeLa cells were assayed for activation of proteins involved in double strand break repair by the homologous recombination-dependent repair pathway [6]. Activation by phosphorylation was observed for ATM and NBN without any dramatic increase in the overall amount of either protein present. Similar studies have confirmed the activation of NBN [16] and added observations showing activation of three other proteins involved in DNA repair, RPA2 [16], FANCD2, and FANCI [20].

A second line of investigation has also suggested that host-encoded DNA repair proteins are involved in HSV1 lytic replication. Host repair proteins were found to be present in HSV1-induced nuclear replication compartments where virus DNA synthesis takes place [23]. Using immunofluorescence light microscopy it was demonstrated that replication compartments contain host repair proteins including: ATR, ATRIP, ATM, CHEK2, RPA2, RP1, RAD51, NBN, XRCC5, MSH2, MSH6, FANCD2, and FANCI [6, 16, 18–20, 24, 25]. A thorough proteomic analysis has also demonstrated the presence of multiple host DNA repair proteins in HSV1 replication compartments [26]. In this study, repair proteins were considered to be present in replication compartments if they were coisolated in immunoprecipitates of pUL29 (ICP8), an HSV1-encoded protein enriched in replication compartments.

### 5.2. Activation of DNA Repair Proteins Potentiates Lytic HSV1 Replication

The enhancing role of DNA repair proteins for HSV1 lytic replication is an essential feature of the DNA repair-centered pathway. Entry into latency is expected to be possible only in cells that are minimally permissive for lytic replication due to a deficiency of repair proteins. Two types of studies have been carried out to test the involvement of repair proteins: (1) lytic replication was measured in mutant cells deficient in the repair protein to be tested. Control infections were performed with the same cells after complementation with a gene encoding the functional protein. Virus replication was expected to be observed in the second condition, but not the first if the protein examined enhances lytic HSV1 replication. (2) Lytic replication was examined in cells where expression of the test protein was suppressed by treatment with specific siRNA. Controls in this case were performed with nonspecific siRNA.

An example of the first type of study was performed with a cell line deficient in FANCA, a protein required for DNA repair and involved in the etiology of Fanconi anemia (FA-A cells; [20]). HSV1 replication was tested in FA-A cells and also in FA-A cells after complementation with a gene encoding wild type FANCA protein. The results demonstrated enhanced virus growth only in the complemented cell line providing evidence that FANCA potentiates HSV1 replication. Similar studies involving deletion and complemented cell lines have demonstrated that efficient HSV1 replication requires DNA repair proteins FANCD2, FANCG, MRE11, and ATM [6, 20].

In studies involving siRNA technology, HSV1 replication is measured in control cells and in cells in which expression of a test DNA repair protein is blocked with a specific siRNA. Such studies require a control in which it is demonstrated that the specific siRNA actually depletes test cells of the target repair protein. In a representative study, HFF-1 cells were depleted of RP1 (RPA70), a protein involved in nucleotide excision repair. HSV1 lytic replication was then measured in the depleted cells and in control cells treated with a nonspecific siRNA [19]. Replication was found to be efficient only in control cells indicating that RP1 favors HSV1 growth. Similar studies have demonstrated that efficient HSV1 lytic replication requires proteins involved in mismatch repair and in ATR repair pathway proteins [18, 19].

### 5.3. DNA Repair Proteins Are Not Activated following HSV1 Infection of Neurons

The failure of neurons to respond to HSV1 infection by activating the DNA damage response is documented in an elegant experiment from the Weitzman lab [6]. The study was carried out with a pluripotent human embryonic stem cell line (Cyth25) that can be induced to differentiate in culture into neurons [27]. HSV1 replication and DNA repair protein (ATM) activation were compared in both the undifferentiated and fully differentiated neuron forms of Cyth25. The results showed that efficient virus replication and repair protein activation occurred only in the undifferentiated form of Cyth25 cells. Neurons were negative in both tests supporting the view that they are well suited to serve as hosts for latent HSV1 infection.

It is relevant to note here that neurons have an additional property that makes them attractive as hosts for latent HSV1. Even in the absence of virus infection, neurons are found to be depleted in overall DNA repair capacity compared to other cell types and also to undifferentiated neuronal precursor cells. Experimental evidences supporting the above conclusions are found in studies involving tests of the ability of neurons to repair damaged virus DNA introduced into the cell [21] and tests of the ability of neurons to repair oxidative damage to their own DNA [22].

### 5.4. Excess of DNA Damage Leads to Reactivation of Latent HSV1 from Neurons

Quite diverse lines of evidence support the idea that reactivation involves mobilization of DNA repair functions. One is the observation described above demonstrating that DNA repair functions are necessary for lytic HSV1 replication. Since reactivation results in cycles of lytic HSV1 replication, it is most reasonable to expect that repair functions will also be required for lytic replication resulting from reactivation. A second line of evidence is the clinical observation familiar to physicians who treat HSV1 infections. Reactivated infections are often found to follow exposure of the patient to sunlight [28]. The ultraviolet component of sunlight has the potential to cause DNA damage that could be the initiating factor. Reactivation could also be driven by natural characteristics of the HSV1 genome that are able to launch DNA repair pathways. Such characteristics include the G quadraplex structures resulting from the high G:C content (68%) of HSV1 DNA [29] and features such as inverted and tandem repeats able to promote recombination-dependent repair [30].

Finally, there are relevant cell culture studies that suggest involvement of repair functions in reactivation. One such study was carried out with a mouse neuroblastoma cell line (C1300; [31]) that replicated HSV1 poorly as expected of a neuronal cell. Virus replication was found to be improved significantly, however, when the cells were treated with agents (such as etoposide and hexamethylene bisacetamide) that cause DNA damage.

## 6. Other Features of Latency Consistent with the DNA Repair-Centered Pathway

It is an attractive feature of the repair-centered pathway that important aspects are compatible with previously known features of HSV1 latency and reactivation. An example is the specificity for latency in neurons. While activation of repair functions is observed in most cell types able to host HSV1 infections, this is not the case with neurons. Here lytic replication is blocked by the absence of activated repair proteins creating an environment conducive to latency. Neurons of the peripheral compared to the central nervous system are especially well suited for a role in latency. The blood brain barrier is found to be more permeable in the PNS [32], a property that favors the availability of small molecules able to promote virus reactivation. Neurons of the PNS are therefore well suited to provide a way for latent HSV1 to escape its confinement in the latent state.

The repair-centered pathway is also compatible with the observation that a high rate of genetic recombination is characteristic of HSV1 genomes during lytic replication. Active recombination has been identified at most sites in the genome with particularly high activity at the junctions of L and S segments [33–36]. The requirement of repair functions for HSV1 lytic replication provides a potential source for the required recombination events. Homologous recombination, for instance, is an integral feature of important repair pathways including the synthesis-dependent strand-annealing pathway of double strand break repair [37]. The high rate of HSV1 genome recombination can therefore be regarded as a consequence of the requirement for DNA repair pathways.

Finally, the genetic identity of infecting and reactivated HSV1 strains also supports the repair-centered pathway. The DNA sequence identity of initial and reactivated virus is often overlooked because it is obvious that such identity must be the rule. Without it there would be no genetic continuity in the HSV1 species. Viewed more closely, however, it is clear that genetic identity is quite a remarkable fact [38]. During the period of latency the HSV1 genome is expected to be subject to the same variety of toxic affects found for all cells. These include, for example, ionizing radiation, environmental mutagenic chemicals, and mutations occurring during DNA replication. If not repaired, these would introduce instability into the virus genome as they do for the cell. The situation is more severe in the case of latent neurons because repair functions are not activated.

The proposed repair-centered pathway suggests a solution by postulating that reactivation is caused by mobilization of DNA repair capacity. A consequence of the model is that repair of DNA damaged during latency is expected to occur as a part of the reactivation process.

## 7. Future Directions

As with most scientific hypotheses, the pathway discussed here for HSV1 latency and reactivation would benefit from future experimental testing. The identity of the repair proteins required for lytic growth is an example. It is clear that not all repair proteins are required [39], but can a minimum subset be defined? The proposed mechanism of reactivation in particular is in need of further evaluation. We need to know more about which repair functions are mobilized as the virus is reactivated. Are they the same as those activated as the virus enters into latency or are there differences? Further knowledge about factors that stimulate reactivation would also be most welcome. The roles of stress and mutagenic effects are now appreciated, but it would be of interest to know more about the specific biochemical signaling agents involved.
